# Identification of germ cell-specific genes in mammalian meiotic prophase

**DOI:** 10.1186/1471-2105-14-72

**Published:** 2013-02-27

**Authors:** Yunfei Li, Debjit Ray, Ping Ye

**Affiliations:** 1 School of Molecular Biosciences, Washington State University, PO Box 647520, Pullman, WA 99164, USA; 2 Biological Systems Engineering, Washington State University, Pullman, WA 99164, USA; 3 Center for Reproductive Biology, Washington State University, Pullman, WA 99164, USA

## Abstract

**Background:**

Mammalian germ cells undergo meiosis to produce sperm or eggs, haploid cells that are primed to meet and propagate life. Meiosis is initiated by retinoic acid and meiotic prophase is the first and most complex stage of meiosis when homologous chromosomes pair to exchange genetic information. Errors in meiosis can lead to infertility and birth defects. However, despite the importance of this process, germ cell-specific gene expression patterns during meiosis remain undefined due to difficulty in obtaining pure germ cell samples, especially in females, where prophase occurs in the embryonic ovary. Indeed, mixed signals from both germ cells and somatic cells complicate gonadal transcriptome studies.

**Results:**

We developed a machine-learning method for identifying germ cell-specific patterns of gene expression in microarray data from mammalian gonads, specifically during meiotic initiation and prophase. At 10% recall, the method detected spermatocyte genes and oocyte genes with 90% and 94% precision, respectively. Our method outperformed gonadal expression levels and gonadal expression correlations in predicting germ cell-specific expression. Top-predicted spermatocyte and oocyte genes were both preferentially localized to the X chromosome and significantly enriched for essential genes. Also identified were transcription factors and microRNAs that might regulate germ cell-specific expression. Finally, we experimentally validated *Rps6ka3*, a top-predicted X-linked spermatocyte gene. Protein localization studies in the mouse testis revealed germ cell-specific expression of RPS6KA3, mainly detected in the cytoplasm of spermatogonia and prophase spermatocytes.

**Conclusions:**

We have demonstrated that, through the use of machine-learning methods, it is possible to detect germ cell-specific expression from gonadal microarray data. Results from this study improve our understanding of the transition from germ cells to meiocytes in the mammalian gonad. Further, this approach is applicable to other tissues for which isolating cell populations remains difficult.

## Background

Multi-cellular eukaryotes are made of two fundamental cell types—germ cell and somatic cell. The distinguishing characteristic of a germ cell is its capability to undergo meiosis. Meiosis is a highly specialized cell division that converts diploid germ cells into haploid sperm or eggs, cells that are primed to meet for the propagation of the organism. Mammalian meiosis is initiated by an extrinsic signal—retinoic acid—and consists of meiosis I and II, each of which is divided into prophase, metaphase, anaphase, and telophase [1-4]. Prophase of meiosis I (abbreviated as prophase) is the first and most complex stage of meiosis, when maternal and paternal homologs pair to allow the exchange of genetic information. Based on chromosomal packaging, prophase itself is subdivided into five stages: leptotene, zygotene, pachytene, diplotene, and diakinesis.

Although the components of meiosis are similar in both sexes—pre-meiotic germ cell proliferation and differentiation, initiation and progression through meiosis, and gamete maturation—the fundamentals are dramatically different with respect to timing, outcome, and ability to produce normal gametes [5]. The first wave of spermatogenesis begins in puberty and proceeds relatively synchronously, which is followed by continuous and asynchronous spermatogenesis throughout life. In contrast, initiation of oogenesis is confined to a narrow window of fetal development. The entire pool of oocytes initiates meiosis in a semi-synchronized manner, but the process arrests at the end of prophase, before birth. One arrested oocyte, on average, then resumes oogenesis during each ovulation cycle starting from puberty [3,4,6]. At the end of meiosis, a single egg is produced in females, compared with four sperm in males. An additional difference is that the incidence of aneuploid gametes produced in humans is at least an order of magnitude greater in the female than the male [5].

Meiotic entry and progression require highly precise and ordered gene expression. Identifying these gene expression signatures is imperative to circumvent clinical disorders, including infertility, birth defects, and germ cell tumors. However, our understanding of the factors that control germ cell entry into and progression through meiosis remains rudimentary. This is because studies of mammalian germ cells are usually limited to *in vivo* animal models. Further, oocytes enter meiosis during fetal life, when access to ovarian tissue is extremely limited. While time-series transcriptome studies of mammalian gonads have delineated the temporal sequence of genome-wide expression [7-13], identifying germ cell-specific genes necessary for meiosis has been difficult due to the mixture of germ and somatic cells in gonads, each of which contributes to the total transcriptome. Although it is possible to isolate germ cells from the testis using physical separation methods [14,15], isolation of pure oocyte populations from the fetal ovary has been challenging due to the limited amount of ovarian tissue. Further, gene expression and cell physiology may differ in sorted germ cell samples versus *in vivo* populations, and the purity of isolated samples has been questioned.

Ideally, germ cell expression signals would be deciphered from whole-gonadal expression without physically isolating germ cells. Here, we applied a machine-learning algorithm, support vector machine (SVM), to predict mouse germ cell genes during meiotic initiation and prophase from time-course gonadal microarray profiles. This timeframe was selected for two reasons. First, prophase is the most important and complicated stage of meiosis. Second, the entire germ cell pool progresses through prophase in a relatively synchronized fashion during oogenesis and the first wave of spermatogenesis, thus global gene expression can be monitored by microarrays. Our approach allowed us to locate hidden germ cell patterns at high resolution and outperformed other methods in detecting germ cell-specific expression from mixed gonadal samples. Further, our method ranked genome-wide mouse genes according to the probability of being expressed by germ cells, enabling prioritization of candidate genes for experimental follow-up. In summary, results from this study increase our knowledge of germ cell-specific expression during the critical stage of meiotic initiation and prophase. Predicted germ cell genes advance our understanding of the genetic control of germ cell development, sex-specific differences in meiosis, as well as factors predisposing to infertility and birth defects.

## Results

### Computational models to predict germ cell genes during meiotic initiation and prophase

Germ cells, but not somatic cells, of the testis and ovary undergo meiosis. Microarray profiles of mammalian gonads, however, record combined signals from both germ cells and somatic cells. We built SVM classifiers to predict mouse germ cell genes in meiotic initiation and prophase from gonadal microarray data. SVM identified a combination of expression patterns in the microarray profile that maximally separated genes expressed by germ cells from those not expressed by germ cells. We developed two versions of the SVM classifier: the spermatocyte model predicted germ cell genes using spermatocyte training examples and microarray studies on postnatal testis during prophase of the first wave of spermatogenesis; the oocyte model predicted germ cell genes using oocyte training examples and microarray studies on embryonic ovary during prophase [12,13,16]. Genes known to be expressed by germ cells in prophase served as the positive training set, and genes known not to be expressed by germ cells served as the negative training set. Our positive training data were all derived from single-gene studies [9,12,17-19]. Importantly, the training data were completely independent from the microarray studies, which served as the features of the SVM classifiers.

**Figure 1:**
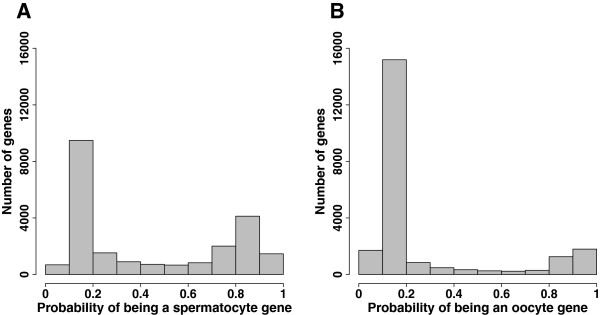
**Germ cell models predict germ cell-specific expression during meiotic initiation and prophase.** Histograms display the predicted probability of being a germ cell gene for genome-wide mouse genes. **A**. Predictions from the spermatocyte model. **B**. Predictions from the oocyte model.

For each gene in the mouse genome, our germ cell models predicted the probability the gene was expressed by germ cells during meiotic initiation and prophase. The probability ranged from 0 to 1, where 0 indicated the gene was not expressed by germ cells, and 1 indicated the gene was expressed by germ cells. We examined the model prediction on mouse genes genome-wide. A clear bimodal distribution was observed for predicted probabilities from both the spermatocyte model and oocyte model: most genes had either high or low probability of being germ cell genes (Figure 1). This demonstrated that our models built upon the training set could predict germ cell expression from analysis of whole-gonad microarray data. The top-predicted spermatocyte genes and oocyte genes are listed in Additional file 1: Tables S1-S2.

### Performance evaluation of the germ cell models

**Figure 2:**
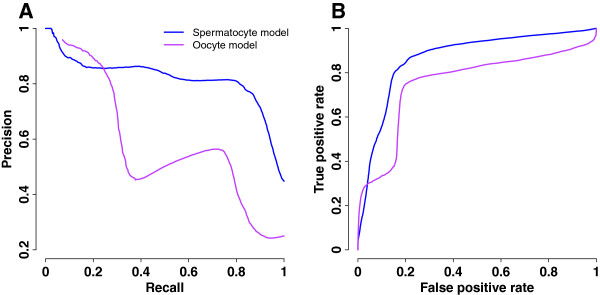
**Performance of germ cell models.** Model performance was evaluated by100 rounds of five-fold cross-validation. Training genes were sorted in descending order of probability of being a germ cell gene, and rates were calculated with a probability decrement of 0.002. **A**. Precision-recall curves. **B**. ROC curves.

Cross-validation is to assess whether a statistical model can be generalized to datasets independent of the training data used for building the model. To evaluate the classification accuracy of our germ cell models, we employed a five-fold cross-validation, whereby 80% of the training examples were used for building the classifier while the remaining 20% were reserved for evaluation and the process iterated five times. We repeated the five-fold cross-validation 100 times, and then sorted genes in the descending order of predicted probability of being a germ cell gene. Precision and recall were computed from the ranked sequence of genes. Precision is the fraction of correctly predicted germ cell genes over predicted germ cell genes. A perfect precision of 1 means that every predicted germ cell gene is true. Recall is the fraction of correctly predicted germ cell genes over all germ cell genes. A recall of 1 means complete coverage of germ cell genes, i.e., all germ cell genes are predicted to be true. We plotted precision-recall curves to display results from cross-validations of the germ cell models (Figure 2A, Table 1).

**Table 1:** Performance comparison between the germ cell models and other methods

	**Spermatocyte gene**			**Oocyte gene**		
	**Precision at 10% recall**	**Average precision**	**Random precision**^**&**^	**Precision at 10% recall**	**Average precision**	**Random precision**
Germ cell model	90%	78%	45%	94%	52%	25%
Gonadal expression level	76%	67%	45%	67%*	59%	25%
Gonadal expression correlation	59%	52%	50%	59%	61%	62%

^&^Random precision was the precision when recall equaled one.

*The value was computed based on linear interpolation.

The precision of identifying a spermatocyte gene at random was 45% based on the training data (129 positive examples and 159 negative examples). The spermatocyte model reached a precision of 90% at 10% recall, a two-fold improvement from random precision. The precision of identifying an oocyte gene at random was 25%, as estimated from the training data (46 positive examples and 138 negative examples). The oocyte model yielded a precision of 94% at 10% recall, close to a four-fold increase from the random precision. Average precisions of 78% and 52% were achieved for the spermatocyte model and oocyte model, respectively, equivalent to 1.7 and 2.1-fold increments of random precisions. The average recall is 68% for the spermatocyte model and 57% for the oocyte model. These results suggest that our models are highly accurate in predicting top-ranked germ cell genes, but not necessarily sensitive in overall classification of germ and non-germ cell genes.

We further evaluated the performance of germ cell models by receiver operating characteristic (ROC) curves (Figure 2B). True positive rate (recall) is the fraction of correctly predicted germ cell genes over all germ cell genes while false positive rate is the fraction of incorrectly predicted germ cell genes over all non-germ cell genes. We observed that the spermatocyte model performed better than the oocyte model based on the area under the ROC curve (AUC=0.87 versus 0.75). However, the lower left portion of the ROC curves indicated comparable performance. For a true positive rate of 10%, the spermatocyte and oocyte models showed a false positive rate of 1% and 0.2%, respectively, suggesting the top-ranked germ cell genes are the most reliable predictions.

### Performance comparison with other prediction methods

**Figure 3:**
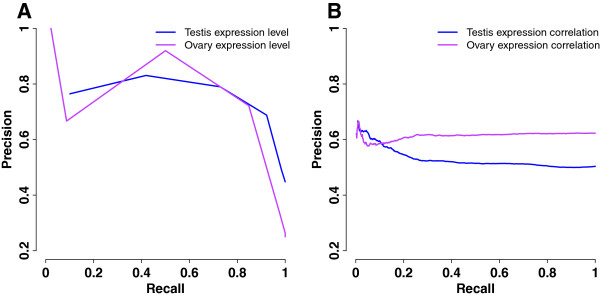
**Alternative methods for predicting germ cell-specific expression from gonadal microarray studies.** Precision-recall curves were plotted to evaluate the performance of alternative methods. **A**. Gonadal expression levels. The average of gonadal expression across prophase was computed for each training gene. Genes were sorted in descending order of log2 expression, and precision and recall were calculated with a log2 expression decrement of 2. **B**. Gonadal expression correlation. Pearson correlation of gonadal expression across prophase was calculated for each training gene pair. Gene pairs of the same type (both germ cell genes or non-germ cell genes) served as the positive correlation examples; gene pairs of different types (one germ cell gene and one non-germ cell gene) served as the negative correlation examples. Training gene pairs were sorted in descending order of correlation coefficient, and precision and recall were calculated with a coefficient decrement of 0.002.

To further assess the performance of the germ cell models, we developed two alternative approaches to identify germ cell genes from microarray studies of whole gonads. The first was to directly extract genes that were preferentially expressed in the gonad. Training genes were ranked by their gonadal expression levels in microarray studies, and precision and recall were computed from this sorted list (Figure 3A, Table 1). At 10% recall, precisions of 76% and 67% were reached for expression levels of the testis and ovary, respectively, equivalent to 14% and 27% precision reductions compared to the corresponding germ cell models. The average precision of testis expression levels was 67%, 11% lower than that of the spermatocyte model. The average precision of ovary expression levels was 59%, 7% higher than that of the oocyte model.

The second approach was to compute Pearson correlation of gonadal expression across prophase for each training gene pair. Pairs of two germ cell genes or two non-germ cell genes were positive correlation examples, while pairs of one germ cell gene and one non-germ cell gene were negative correlation examples. Training gene pairs were sorted in descending order of correlation coefficient, and precision and recall were computed from the sorted list (Figure 3B, Table 1). The results showed that precisions at 10% recall and average precisions were both close to random precisions, suggesting limited power of this method in predicting germ cell genes.

It is also possible to identify new germ cell genes by performing hierarchical clustering on microarray profiles across all time points of meiotic prophase (Additional file 1: Figure S1). Genes specifically expressed in germ cells (positive training data) exhibited a particular expression pattern, which allowed separating them from other genes. The advantage of our germ cell models over hierarchical clustering is that they can prioritize genes for experimental testing based on the probability of being germ cell genes.

### Performance comparison with microarray expression of male germ cell isolates

Although it remains challenging to isolate a very small number of oocytes from the embryonic ovary, techniques have been developed to isolate male germ cells of different stages with reasonable purity. Two published studies performed global expression profiling on spermatogonia and spermatocytes isolated via gravity sedimentation and sequential enzymatic digestion [7,12,20]. Spermatogonia undergo proliferation and differentiation prior to meiotic initiation; pachytene spermatocytes are in the prophase stage. Therefore, we evaluated expression levels of isolated spermatogonia and pachytene cells in predicting spermatocyte genes during meiotic initiation and prophase.

**Figure 4:**
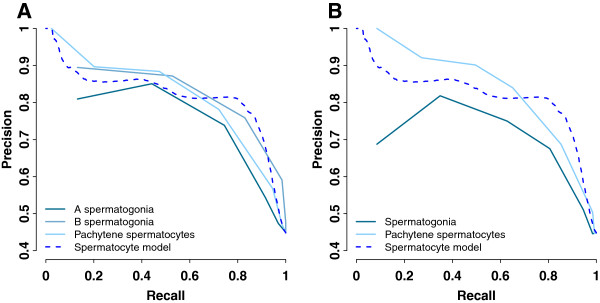
**The spermatocyte model achieves comparable performance to microarray expression of male germ cell isolates.** Precision-recall curves were plotted to evaluate the method performance on classifying training data. The curve of the spermatocyte model in Figure 2A is displayed here again for comparison. **A**. Expression levels of isolated spermatogonia and pachytene spermatocytes from [12,20]. Training genes were sorted in descending order of log2 expression, and precision and recall were calculated with a log2 expression decrement of 2. **B**. Expression levels of isolated spermatogonia and pachytene spermatocytes from [7]. Training genes were sorted in descending order of log2 expression, and precision and recall were calculated with a log2 expression decrement of 2.

Training genes were ranked by their expression levels in germ cell isolates, and precision and recall were computed from this sorted list (Figure 4, Table 2). At 10% recall, pachytene spermatocytes reached higher precision while spermatogonia had lower precision compared to the spermatocyte model. The average precision of the spermatocyte model, however, was superior to any isolate expression. This comparison suggests that SVM prediction of germ cell genes from whole-gonadal expression achieved comparable performance to the expression of germ cell isolates, but without going through the tedious experimental isolation procedure.

**Table 2:** Performance comparison between the spermatocyte model and microarray expression of male germ cell isolates

	**Precision at 10% recall**	**Average precision**	**Random precision**^**&**^
Spermatocyte model	90%	78%	45%
A spermatogonia [12,20]	81%*	62%	45%
B spermatogonia [12,20]	90%*	64%	45%
Pachytene spermatocytes [12,20]	96%*	69%	45%
Spermatogonia [7]	69%*	62%	45%
Pachytene spermatocytes [7]	99%*	72%	45%

^&^Random precision was the precision when recall equaled one.

*The value was computed based on linear interpolation or extrapolation.

### Characterization of predicted germ cell genes

Our models assigned probabilities to mouse genes genome-wide, allowing prioritization of potential germ cell genes for analysis. We focused on the top-1,000 predicted spermatocyte genes and oocyte genes; limited overlap existed between the two gene lists (144 genes, Jaccard index=0.08). We first identified the chromosome location of predicted germ cell genes. Strikingly, both top spermatocyte and oocyte genes were significantly enriched on the X chromosome, and the enrichment was more significant in the female than the male (P-value=0.04 for spermatocyte genes; P-value=0.0002 for oocyte genes). No enrichment was observed in any other chromosome: 1–19 and Y.

We performed the gene ontology (GO) enrichment analyses to characterize the function of the top-1,000 germ cell genes (Table 3). These genes had annotations consistent with the germ cell function during meiotic initiation and prophase. GO terms directly relevant to meiosis included “meiosis”, “mitosis”, “cell cycle”, and “cell division”. Recombination is the hallmark event of prophase, in which two homologous chromosomes pair and exchange genetic information through DNA double strand breaks and repairs. Recombination-relevant GO terms consisted of “response to DNA damage stimulus” and “DNA repair”. Other GO terms described general transcriptional and translational regulation, including “mRNA transport”, “mRNA processing”, “chromatin modification”, “protein transport”, “regulation of translation”, and “ubiquitination”.

**Table 3:** Significantly enriched GO terms among 1,000 top-predicted germ cell genes

**GO id**	**GO name**	**P-value**
*Spermatocyte model*		
GO:0051028	mRNA transport	2.97×10^-3^
GO:0006974	response to DNA damage stimulus	5.83×10^-3^
GO:0006417	regulation of translation	0.01
GO:0007049	cell cycle	0.01
GO:0007126	meiosis	0.02
*Oocyte model*		
GO:0007049	cell cycle	7.22×10^-10^
GO:0015031	protein transport	1.16×10^-9^
GO:0051301	cell division	1.65×10^-7^
GO:0007067	mitosis	5.47×10^-7^
GO:0016192	vesicle-mediated transport	9.07×10^-7^
GO:0006886	intracellular protein transport	4.58×10^-6^
GO:0016568	chromatin modification	7.27×10^-5^
GO:0006281	DNA repair	0.01
GO:0006397	mRNA processing	0.01
GO:0006810	transport	0.01
GO:0006511	ubiquitin-dependent protein catabolic process	0.01
GO:0006974	response to DNA damage stimulus	0.02
GO:0051028	mRNA transport	0.02
GO:0070936	protein K48-linked ubiquitination	0.02

**Figure 5:**
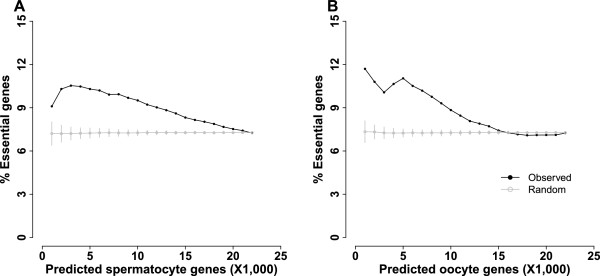
**Essential genes are enriched among top-predicted germ cell genes.** To produce the observed curve, genome-wide genes were sorted in descending order of predicted probability of being a germ cell gene. The fraction of essential genes was computed with an increment of 1,000 genes in the ranked sequence. To produce the random curve, genome-wide genes were randomized and the fraction of essential genes was computed with an increment of 1,000 genes. Randomization was repeated 100 times, and the average and standard deviation were displayed. **A**. Predictions from the spermatocyte model. **B**. Predictions from the oocyte model.

Essential genes are required for mouse viability. We determined whether predicted germ cell genes in meiotic prophase were enriched for essential genes. Mouse essential genes were obtained from the database of Mouse Genome Informatics based on the phenotype of homozygous knockouts (embryonic, prenatal, perinatal, and postnatal lethality) [21]. There were 1,645 essential genes among 22,409 genes genome-wide. Thus, the chance of identifying an essential gene at random was 7%. Genome-wide genes were ranked in descending order of probability of being a germ cell gene, and the percentage of essential genes was computed with an increment of 1,000 genes. As a control, we randomized the order of genome-wide genes and again calculated the percentage of essential genes with an increment of 1,000 genes (Figure 5). We found the top genes predicted from both the spermatocyte model and the oocyte model were enriched for essential genes. The fraction of essential genes was 9% for the top-1,000 spermatocyte genes and 12% for the top-1,000 oocyte genes. The percentage of essential genes decreased with the inclusion of more genes along the sorted list and eventually reached 7%, the random level of essential genes. In contrast, the fraction of essential genes stayed constant at 7% for the randomized list of genes. The fraction of essential genes was significantly higher in the sorted list than the randomized list for both the spermatocyte model and oocyte model (P-value=1.18×10^-7^ for spermatocyte genes; P-value=0.0001 for oocyte genes, one-tailed *t*-test).

### Potential transcription factors activating predicted germ cell genes

Meiotic initiation and progression through prophase depends on a robust, germ cell-specific transcription program. However, the transcription factors for prophase genes remain uncharacterized. Here, we uncovered putative transcription factors by detecting over-represented sequence motifs in the promoter regions of the top-1,000 predicted germ cell genes using FIRE [22].

**Figure 6:**
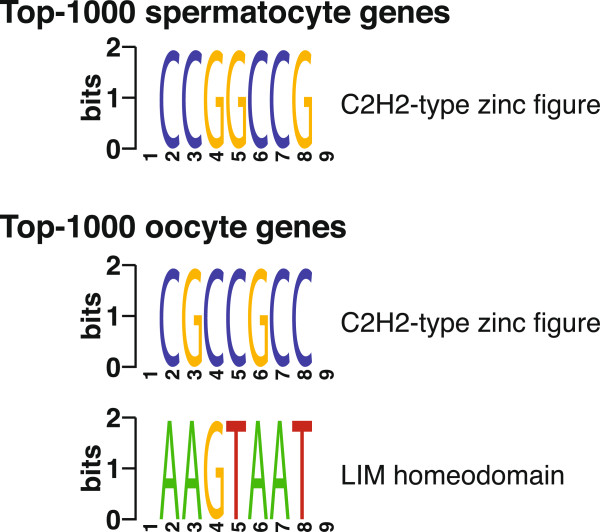
**Over-represented sequence motifs in the promoter regions of top-predicted germ cell genes.** Motifs were detected by FIRE using default parameters. Motif names were labeled based on the closest transcription factor family in JASPAR or TRANSFAC.

One CG-rich motif was significantly enriched among the top-1,000 spermatocyte genes (Figure 6). This motif was identified as the binding site for C2H2-type zinc finger domains. The closest matching transcription factor was *Hinfp*, which activates histone H4 gene transcription at the G1/S phase transition [23]. *Hinfp* was highly expressed in prophase of spermatogenesis, but its function during germ cell development has never been reported.

One CG-rich motif was also significantly over-represented among the top-1,000 oocyte genes. Although this motif was different from the one enriched among top spermatocyte genes, it was also recognized as the binding site for C2H2-type zinc finger domains; both motifs had a position bias towards transcription start sites (Figure 6). *Sp1* was the transcription factor most closely associated with the oocyte CG-motif. *Sp1* binds to CG-rich motifs and regulates the expression of a large number of genes involved in a variety of processes. In particular, *Sp1* mediates transcriptional activation of male germ cell genes that are expressed during meiotic initiation and prophase [24,25]. One LIM homeodomain motif was also enriched among the top-1,000 oocyte genes, and exhibited a strong positive co-occurrence with the CG-rich motif. LIM-homeodomain proteins play fundamental roles in tissue patterning and differentiation [26]. Although *Lhx3* was identified as the best matching transcription factor for this motif, it was barely expressed in the embryonic ovary and had no association with germ cell development. Instead, *Lhx9* is known to express in the embryonic ovary and is essential for mouse gonad formation [27]. Our results suggest that *Lhx9* might be a potential regulator for oocyte genes during meiotic initiation and progression.

### Potential microRNAs repressing predicted non-germ cell genes

Like transcription factors, microRNAs have emerged as critical developmental regulators. MicroRNAs are small endogenous RNAs that typically bind their target 3’UTRs through exact or near-exact complementarity. This binding event leads to translational repression and mRNA degradation of target genes. We were interested in identifying microRNAs that could potentially repress non-germ cell genes predicted from the models.

We obtained target genes of mouse microRNAs from two databases that used distinct prediction algorithms: TargetScanMouse and miRanda [28,29]. Our germ cell models assigned probabilities of prophase expression to genome-wide mouse genes. Thus, for each microRNA, we calculated the area under the ROC curve to evaluate whether the targets of the microRNA were predictive for non-germ cell genes. In this way, we identified four and five microRNAs from the spermatocyte model and oocyte model, respectively, that may mediate the repression of non-germ cell genes (Table 4). The expression and function of most of these microRNAs have not been characterized. All these microRNAs are located on autosomes except mmu-miR-351, which was X-linked and predicted to inhibit oocyte genes. Studies have revealed that mmu-miR-351 was ubiquitously expressed in many adult mouse tissues, including the testis and ovary. In particular, it was expressed in isolated spermatogonia, suggesting a potential function in regulating mRNAs during early stage of spermatogenesis [30]. Our result indicates that mmu-miR-351 might play a conserved role in pre-meiotic oocytes.

**Table 4:** MicroRNAs potentially repressing predicted non-germ cell genes

**MicroRNA**	**Chromosome location**
*Spermatocyte model*	
mmu-miR-615	15
mmu-miR-592	6
mmu-miR-882	12
mmu-miR-185	16
*Oocyte model*	
mmu-miR-615	15
mmu-miR-491	4
mmu-miR-326	7
mmu-miR-330	7
mmu-miR-351	X

An AUC value was calculated for each microRNA in TargetScanMouse (Release 6.1 March 2012, conserved targets of conserved microRNAs) [28] and miRanda (August 2010, targets with good mirSVR score of conserved microRNAs) [29] to evaluate whether microRNA targets overlap with predicted non-germ cell genes. Top-10 microRNAs based on AUC values were obtained from each of the two databases. The microRNAs identified from both databases are shown in the table.

### Experimental validation of predicted germ cell genes

Our models predicted preferential localization of germ cell genes on the X chromosome. We further focused on X-linked spermatocyte genes because functional characterization of knockout mice was relatively easy. Males have one copy of X-linked genes, thus the phenotype of loss-of-function mutations would not be masked by a second allele. Among top-1,000 spermatocyte genes, 43 were X-linked and unique to the male, i.e., not overlapping with top-1,000 oocyte genes. We manually went through the list to identify candidates that were not previously linked to spermatocyte expression and function. *Rps6ka3* (ribosomal protein S6 kinase alpha-3) emerged as an interesting candidate because it encodes a growth-factor-regulated protein kinase and is a known disease gene for which knockout mouse lines and commercial antibodies are available [31-33]. Mutations in this gene are responsible for Coffin–Lowry syndrome, which is characterized in male patients by mental retardation, growth retardation, and skeletal anomalies. The estimated incidence is 1:50,000 to 1:100,000 [34]. In addition, our previous co-expression study also identified *Rps6ka3* as a candidate prophase gene [35].

**Figure 7:**
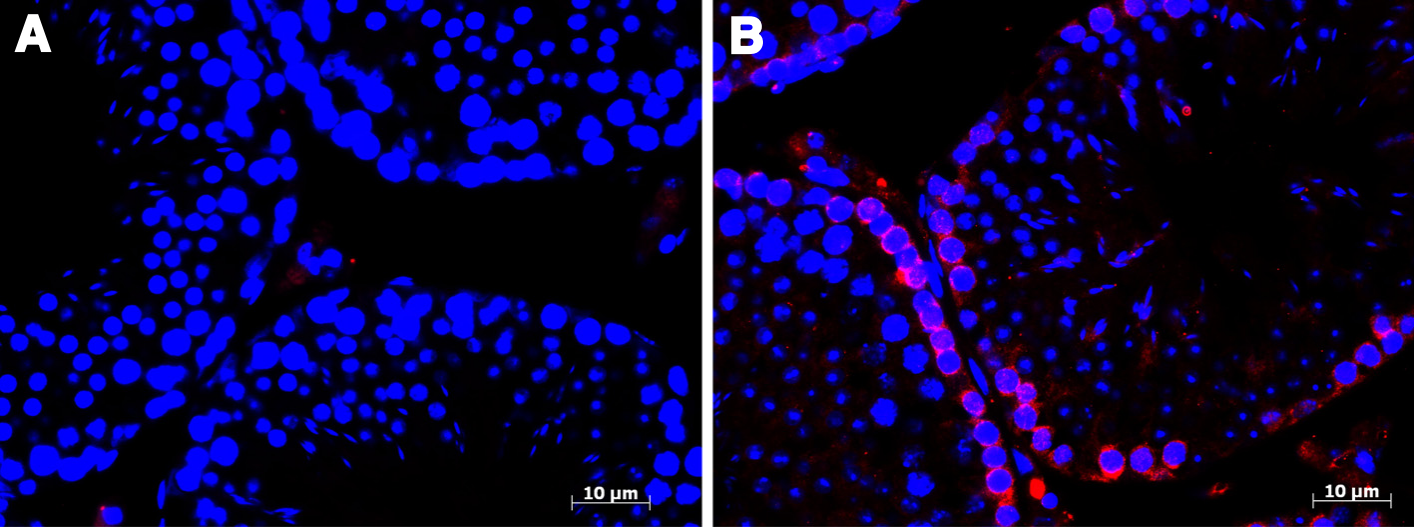
**Experimental validation of RPS6KA3 in the mouse testis.** Immunofluorescence was performed on cross sections of adult mouse testis. DNA was stained blue with DAPI. **A**. No RPS6KA3 antibody served as the control. **B**. RPS6KA3 expression was colored red.

To verify *Rps6ka3* as a spermatocyte gene, we performed immunofluorescence on cross-sections of adult mouse testis using commercial RPS6KA3 antibody [33,36]. We found RPS6KA3 expression was germ cell-specific; no expression was detected in the gonad interstitium or in somatic cells (Figure 7). Protein signals were localized to mitotic and meiotic prophase cells including spermatogonia and leptotene and pachytene spermatocytes; no signal was detected in round and elongated spermatids. Protein expression was mainly confined to the cytoplasm of germ cells. This experiment provided “proof of concept” data, supporting our prediction of *Rps6ka3* as a spermatocyte gene expressed during meiotic initiation and prophase.

## Discussion

Germ cells initiate meiosis in response to the extrinsic factor retinoic acid. Meiotic initiation is followed by prophase, a critical developmental stage of germ cells when homologous chromosomes undergo recombination to generate genetic diversity in offspring. Examining patterns of gene expression at a genomic level is necessary to better understand the process of meiotic initiation and progression as well as to identify key factors involved in the process. Further, comparison of male and female expression time courses allows for better understanding of the sexually dimorphic aspects of germ cell differentiation that may contribute to the inherently high meiotic error rate in the female [5]. Microarrays have been utilized extensively in transcriptome profiling of mammalian gonads [7-13]. A major complication, however, is that the mRNA expression represents a combination of signals from both germ cells and somatic cells.

To overcome this obstacle, we outlined a framework for determining germ cell expression during meiotic entry and progression through prophase from gonadal microarray data in male and female mice. SVM was used to detect hidden patterns of germ cell signals and did not require cell-type frequency in gonadal samples. Our germ cell models accurately predicted spermatocyte genes with a 90% precision and oocyte genes with a 94% precision at 10% recall. Further, our models outperformed other methods substantially in predicting germ cell genes from whole-gonadal expression studies. Although experimental methods have been developed to isolate mRNA samples enriched for spermatogonia and spermatocytes [14,15], oocyte sorting from the embryonic ovary is not yet feasible. It remains a challenge to examine gene expression in embryonic oocytes. Therefore, our study is particularly valuable for identifying oocyte genes from the ovary microarray data.

We have demonstrated that top-predicted germ cell genes had GO annotations consistent with gonadal tissue in prophase. Top-predicted germ cell genes were also significantly enriched for essential genes. This suggests that many genes expressed during meiotic initiation and prophase are essential for mouse viability. One interesting observation is that top-predicted germ cell genes were preferentially located to the X chromosome, but not to any other chromosome. The enrichment on the X chromosome was more significant in the female than in the male. This observation was in strong concordance with sex chromosomal dynamics in pre-meiotic and meiotic stages. In the male, both X and Y chromosomes are active in spermatogonia prior to meiotic entry. In fact, spermatogonium-expressed genes are more densely populated on the X chromosome in both human and mouse [18,19,37]. The X and Y chromosomes continue to be active entering prophase but become transcriptionally silenced at the pachytene stage, a process called meiotic sex chromosome inactivation. This inactivation is mainly driven by the unpaired state of the sex chromosomes [38]. In contrast, in the female, one X chromosome is usually silent due to gene dosage compensation. The inactive X chromosome is reactivated preceding meiotic entry such that both X chromosomes remain active throughout meiosis [39,40]. Therefore, we expected to observe more significant enrichment of germ cell genes on the X chromosome in the female during meiotic initiation and prophase. The characterization of top-predicted germ cell genes suggests that our models are truly informative for germ cell-specific expression.

The use of regulatory sequence motifs to identify potential transcription factors has been successfully applied in many areas [41-43]. We located sequence motifs in the promoter region of top-predicted germ cell genes to determine putative regulators. A C2H2-type zinc finger domain was enriched among both spermatocyte genes and oocyte genes. In addition, a LIM homeodomain was identified only for top oocyte genes. The use of sequence motifs to locate regulators has its limitations: the presence of a motif does not necessarily represent the binding and/or functional activity of a transcription factor, and binding motifs of many transcription factors are unknown. Nevertheless, this approach can serve as an initial screen for potential transcription factors.

Transcriptional regulation of germ cell genes is further complicated by microRNAs [44]. For example, the expression of the Let-7 family of microRNAs is increased in spermatogonia after treatment with retinoic acid [45]. Testis-specific microRNAs are preferentially mapped to the X chromosome and most of the X-linked microRNAs are expressed in pachytene spermatocytes, suggesting possible roles in post-transcriptional regulation of prophase genes [30,46]. In contrast to the male, studies of microRNAs in prophase oocytes are scarce [47]. Based on predicted targets of microRNAs and our germ cell model predictions, we identified several candidate microRNAs that may repress gene expression during meiotic initiation and progression, including the X-linked mmu-miR-351.

Using the germ cell models, we were able to rank genome-wide genes and make high-quality predictions for genes expressed during meiotic initiation and prophase. We were particularly interested in X-linked spermatocyte genes because loss-of-function mutations can be easily obtained by deleting one copy of X-linked genes in the male. We experimentally validated *Rps6ka3*, an X-linked disease gene previously unknown to have meiotic function, in the mouse testis using immunofluorescence. Protein expression was germ cell-specific and was mainly confined to spermatogonia and spermatocytes in prophase, concordant with the model prediction. Thus, this experiment lays a foundation for future meiotic functional study of *Rps6ka3* by characterizing knockout mouse lines [31-33]. Further, this validation experiment serves as a proof of concept and indicates that our systems biology approach integrating computation and experimentation is valuable in the identification of novel meiotic genes. Such large-scale, unbiased, and quantitative studies provide an essential complement to the traditional reductionist approaches by studying individual genes.

## Conclusions

Results from this study provide a fundamental understanding of germ cell genes active in meiotic initiation and prophase, a critical developmental stage. We have demonstrated that, through the use of machine-learning methods, it is possible to detect germ cell-specific signals from gonadal microarray datasets. Our ability to make such predictions will likely improve with the increased number of germ cell genes being characterized in the future. While we are primarily motivated by meiotic prophase studies of germ cells, this approach is applicable to a variety of areas in which it is not yet possible to obtain pure cell samples [48-50].

## Methods

### Training data of germ cells

Our goal was to predict germ cell genes expressed during meiotic initiation and prophase in male mouse and female mouse. Thus, positive training examples were genes currently known to express while negative training examples were genes currently known not to express in prophase of germ cells. We obtained the training data from the literature and the mouse Gene Expression Database (GXD) [17]. GXD collects detailed single-gene expression data from RNA *in situ* hybridization, immunohistochemistry, Northern and Western blots, RT-PCR, RNase and nuclease S1 protection assays, and *in situ* knock-in reporters. Genes that are expressed and not expressed in an anatomical structure and a developmental stage are recorded in the database. Genes labeled as “Very strong”, “Strong”, and “Present” were collected as positive training data; genes labeled with “Absent” and “Trace” were negative training data. Genes with conflict assignments were treated as positive examples. Note that the database does not include any large-scale expression studies (i.e., microarray).

The male training data included genes studied in spermatogonia and primary spermatocytes during postnatal development, as defined in GXD [17]. Additionally, we collected male training data from the literature: genes expressed in premeiotic and prophase germ cells as positive examples [9,12,18,19], and genes only expressed in Leydig, Sertoli, and Myoid cells as negative examples [12]. A total of 137 positive and 26 negative germ cell genes were obtained for the male mouse. Similarly, we collected female training data from primordial germ cells and primary oocytes in the fetal ovary during embryonic days 12–16, recorded in GXD [17]. Training data were further supplemented with genes manually curated from the literature [9]. In total, 47 positive and 4 negative examples served as the training data for the female mouse.

Because of the limited number of negative training examples, we also obtained negative data from microarray profiles of 61 mouse tissues [51]. Genes only expressed in one tissue type, except testis or ovary, were collected. Specifically, the dataset across all tissue types was concatenated, and the median expression value was extracted. Tissue-specific genes were those exhibiting more than 10-fold of median expression value in one tissue except testis or ovary but showing less than the median expression in other tissues. In this way, we obtained 177 tissue-specific genes as negative training examples for both males and females. We combined these genes with those from single-gene experiments, and further limited the training data to those present in the Mouse Genome 430 2.0 Array (Affymetrix, Santa Clara, CA). Finally, a total of 288 genes served as the training data (129 positive and 159 negative examples) for the spermatocyte model and 184 genes served as the training data (46 positive and 138 negative examples) for the oocyte model.

### Microarray data on gonadal tissue and male germ cells

Time-series microarray studies have been conducted to characterize global gene expression in the mouse testis and ovary during germ cell progression through meiotic prophase (GSE12769 and GSE6916) [12,13,16]. In these published studies, whole testes were obtained from male mice at postnatal days 6, 8, 10, and 14 during the first wave of spermatogenesis; whole ovaries were collected from female mice at embryonic days 11.5, 12.5, 14.5, and 16.5. Expression values at each of the time points served as the features for the SVM classifiers. In both testis and ovary studies, duplicate samples were obtained and applied to Mouse Genome 430 2.0 Array (Affymetrix, Santa Clara, CA). The raw data were normalized by MAS5 and signals from duplicate samples were averaged. The probe-sets were translated into genes based on NetAffx Annotation Release 31. The expression level of each gene was defined by choosing the value from the top level probe-set as ranked by Affymetrix. In case of more than one probe-set present at the top level, the average value was used.

Two published studies described global gene expression of isolated male germ cells. One study isolated type A and B spermatogonia, and pachytene spermatocytes via gravity sedimentation; the purity of spermatogonia was >85% and the purity of pachytene spermatocytes was >95% [12,20]. The other study isolated spermatogonia and pachytene spermatocytes via sequential enzymatic digestion and sedimentation unit gravity; spermatogonia were obtained at a purity >85% and pachytene spermatocytes were obtained at a purity >82.5% [7]. In both cases, duplicate samples were obtained and applied to Mouse Genome 430 2.0 Array (Affymetrix, Santa Clara, CA). Data processing followed the same procedure as analyzing gonadal microarray data.

### SVM classifiers to predict germ cell genes

We built SVM classifiers to predict genes expressed by germ cells during meiotic initiation and prophase using the e1071 package in R [52]. Given a set of training genes known expressed or not expressed by germ cells, SVM classifiers identified a specific pattern of expression from microarray experiments that could best separate germ cell genes from non-germ cell genes. Specifically, SVM classifiers are trained through minimizing $\frac{1}{2}\beta^{T}\beta +C\sum_{i=1}^{N}\zeta_{i}$, which is subject to *y*_*i*_(*β*^*T*^*ϕ*(*x*_*i*_) + *β*_0_) ≥ 1 − *ζ*_*i*_, *ζ*_*i*_ ≥ 0. Here, *N* is the number of training examples, *x*_*i*_ is the vector of microarray data on training example *i*, *x*_*i*_ is mapped into a higher dimensional space by the function *ϕ*(*x*_*i*_), *β*^*T*^*ϕ*(*x*_*i*_) + *β*_0_ is the discriminant function to determine the classification of *y*_*i*_ (*y*_*i*_=1 or −1), *ζ*_*i*_ is the slack variable, and *C* is the penalty parameter. The kernel function, *K*(*xi*, *xj*) = *ϕ*(*x*_*i*_)^*T*^*ϕ*(*x*_*j*_), measures the similarity between two training examples, *i and j*. Different kernel functions were explored, including linear, polynomial, sigmoid, and radial basis function. Parameters for each kernel were empirically optimized on the training set through a grid search to achieve the best performance. Classifiers with the best parameters were evaluated by five-fold cross-validation, which was repeated 100 times. Based on AUC values of ROC curves from cross validation, SVM classifiers with a radial basis kernel performed best for both the spermatocyte model and oocyte model. The radial basis kernel is defined by *K*(*xi*, *xj*) = exp(−*γ*‖*xi* − *xj*‖^2^), where *γ* is the kernel width. The optimal parameters for the spermatocyte model are *γ=*8 and *C*=2; the optimal parameters for the oocyte model are *γ*=1 and *C*=2048.

### Chromosome localization

Chromosome localization of mouse genes was based on the UCSC genome annotation database for the December 2011 assembly of the mouse genome (GRCm38/mm10). To determine whether top-1,000 predicted genes have preferential chromosome location, we computed a hypergeometric  P-value: $P\left(X\geq k\right)=\sum_{X=k}^{\min\left(m,n\right)}\frac{\left(\begin{array}{l} m\\ k \end{array}\right)\left(\begin{array}{l} N-m\\ n-k \end{array}\right)}{\left(\begin{array}{l} N\\ n \end{array}\right)}$, where *N* is the number of mouse genes genome-wide, *m* equals 1,000, the number of top-predicted germ cell genes, *n* is the number of genes located on a chromosome, *k* is the number of top predicted genes and located on the chromosome. To correct for multiple hypothesis testing, the P-value of chromosome enrichment was further subjected to Bonferroni correction. We considered that the top-1,000 predicted genes were significantly enriched on a chromosome if and only if *P*(*X*≥*k*)<0.05*/M*, where *M*=21, the number of chromosomes (1–19, X and Y) in the mouse.

### GO term enrichment

Full ontology file (V1.2) and mouse gene association file (V1.919) were downloaded from http://www.geneontology.org/. To identify GO terms significantly enriched among top-1,000 predicted germ cell genes, we computed a hypergeometric P-value with the same formula as chromosome localization but different notations as follows: *n* is the number of genes annotated by a GO term and *k* is the number of top-1,000 predicted genes and annotated by the GO term. The P-value of GO term enrichment was corrected for multiple testing by multiplying with the number of GO terms considered.

### Sequence motifs

We used FIRE, a motif finding algorithm [22], to search for over-represented motifs in the promoter regions of predicted germ cell genes. The promoter region was obtained from the UCSC Genome Browser (GRCm38/mm10) and included 8 kb upstream and 2 kb downstream of characterized transcription start site. Exons and repetitive sequences were masked for motif searching. Motifs were present on either transcribed or non-transcribed strand. Potential transcription factors were identified by comparing motifs to known binding sites of mammalian transcription factors in JASPAR and TRANSFAC databases [53,54] using STAMP, a tool for DNA motif matching [55].

### Immunofluorescence experiments

All animal procedures have been approved by the Washington State University Animal Care and Use Committee. The BL/6-129 mice were housed in a specific-pathogen-free facility. Adult males around 90 days postpartum were euthanized by exposure to a highly concentrated atmosphere of CO_2_ and testicular tissue was collected. Tissue was fixed with 4% paraformaldehyde, and subsequently dehydrated with ethanol and embedded in paraffin. Tissue sections of 4 μm were placed on slides for immunofluorescence experiments [56].

Tissue slides were boiled in 0.01 M citrate buffer (pH 6) for 5 min, then blocked with 10% donkey serum for 30 min. Incubation with goat RPS6KA3 antibody (1:100; sc-1430, Santa Cruz Biotechnology) was performed at room temperature overnight [33,36]. Tissue sections were subsequently incubated with Alexa Fluor 568 donkey anti-goat IgG (1:1,000; Invitrogen) at room temperature for 1 h. Tissue slides were mounted by ProLong® Gold Antifade Reagent with DAPI (Invitrogen) and digitally photographed using a Zeiss Axioplan microscope. Control experiments followed the same procedure except incubation without RPS6KA3 antibody. Cross-sections of testis from at least three mice were analyzed.

## Competing interests

The authors declare that they have no competing interests.

## Authors’ contributions

YL, DR, and PY carried out the studies. PY conceived of the study and drafted the manuscript. All authors read and approved the final manuscript.

## Supplementary Material

Additional file 1: Table S1 Top-100 predicted spermatocyte genes. **Table S2.** Top-100 predicted oocyte genes. **Figure S1.** Global gene expression and training data expression in meiotic prophase. Gene expression levels were log2 transformed. The global expression data were analyzed by average linkage hierarchical clustering using uncentered correlation as distance metrics. The average expression profiles of training data are shown at the bottom of the figure. Click here for file
