# Supplementary material for: Identification of germ cell-specific genes in mammalian meiotic prophase
Source: BMC Bioinformatics. 2013 Feb 27;14:72. doi: 10.1186/1471-2105-14-72 (PMC3599307; doi:10.1186/1471-2105-14-72)
Supplement: Additional file 1: Table S1 — Top-100 predicted spermatocyte genes. Table S2. Top-100 predicted oocyte genes. Figure S1. Global gene expression and training data expression in meiotic prophase. Gene expression levels were log2 transformed. The global expression data were analyzed by average linkage hierarchical clustering using uncentered correlation as distance metrics. The average expression profiles of training data are shown at the bottom of the figure. [file 1471-2105-14-72-S1.pdf]

Additional file 1

Identification of germ cell-specific genes in mammalian meiotic prophase

Yunfei Li[1], Debjit Ray[1,2], Ping Ye[1,3§]

[1]School of Molecular Biosciences, [2]Biological Systems Engineering, [3]Center for Reproductive Biology, Washington State University, PO Box 647520, Pullman, WA 99164, USA

[§]Corresponding author

Email addresses:

YL: yunfei_li@wsu.edu

DR: debjit_ray@wsu.edu

PY: pye@wsu.edu

Table S1 - Top-100 predicted spermatocyte genes

Rank	Mouse gene symbols
1	*Zfp541*
2	*Stk33*
3	*1700006A11Rik*
4	*Syngr4*
5	*5330409N07Rik*
6	*Prps1l1*
7	*Gpr160*
8	*1700123I01Rik*
9	*LOC100047259*
10	*Prss50*
11	*Mdh1b*
12	*Cmtm2a*
13	*Lypd4*
14	*Ttc30a2*
15	*Spesp1*
16	*Morc2b*
17	*1700066D14Rik*
18	*Zmynd10*
19	*BC051142*
20	*Pacrg*
21	*Dynlrb2*
22	*1700006J14Rik*
23	*9330102E08Rik*
24	*1700003M02Rik*
25	*1700013G23Rik*
26	*Gm3925*
27	*Dmrtc2*
28	*Acsl6*
29	*1700074P13Rik*
30	*2900006K08Rik*
31	*Adam5*
32	*Glipr1l2*
33	*4930519N06Rik*
34	*Tekt1*
35	*Gtf2a1l*
36	*1700097M23Rik*
37	*Ccdc158*
38	*Gm10345*
39	*1700029P11Rik*
40	*Spink2*
41	*Msh4*
42	*1700010I14Rik*
43	*4930549O18Rik*

44	*Eid3*
45	*Slc41a2*
46	*Boll*
47	*Tcam1*
48	*4930556A12Rik*
49	*Inca1*
50	*1700012B09Rik*
51	*4930405N21Rik*
52	*Cetn4*
53	*Gsto2*
54	*Lrrc34*
55	*AW552889*
56	*4930444F02Rik*
57	*Ptpn20*
58	*Mlc1*
59	*1700027A23Rik*
60	*Hdgfl1*
61	*Ccnb3*
62	*Pet2*
63	*4930463O16Rik*
64	*Nme5*
65	*Cep72*
66	*4930511M11Rik*
67	*Cetn1*
68	*Drd4*
69	*Bhmt*
70	*1700028J19Rik*
71	*Efhc1*
72	*1700040L02Rik*
73	*Ttll8*
74	*Ccdc18*
75	*Sfrs14*
76	*4922502D21Rik*
77	*1700016M24Rik*
78	*Ncrna00086*
79	*Actn3*
80	*Lrrc50*
81	*Lrp8*
82	*Anxa9*
83	*Tmem87a*
84	*Phyhipl*
85	*Ttc26*
86	*Ero1lb*
87	*Fbxo15*
88	*Pou6f1*
89	*Arap2*

90	*Corin*
91	*Gm884*
92	*Hsf2bp*
93	*Rimklb*
94	*Cep290*
95	*Spo11*
96	*Zfp385b*
97	*1700007L15Rik*
98	*Pfn4*
99	*Asrgl1*
100	*4633401B06Rik*

Table S2 - Top-100 predicted oocyte genes

Rank	Mouse gene symbols
1	*Pde6d*
2	*Rab18*
3	*Ccdc79*
4	*Phf16*
5	*Btbd10*
6	*LOC100047893*
7	*Tm9sf2*
8	*Nupl1*
9	*Stx12*
10	*Mpp5*
11	*Igf1*
12	*Rab11a*
13	*Uimc1*
14	*Pank3*
15	*AW549877*
16	*Smc3*
17	*Gpat2*
18	*Pdk3*
19	*Irs1*
20	*D6Mm5e*
21	*Ciao1*
22	*Csrp1*
23	*Usp9x*
24	*Rraga*
25	*Osbpl2*
26	*Sun1*
27	*Ythdc1*
28	*BC005537*
29	*Cstf2*
30	*Cmpk1*
31	*Eif2b3*
32	*Bclaf1*
33	*Thumpd1*
34	*Asf1b*
35	*Rchy1*
36	*Dnm1l*
37	*Tpr*
38	*Jagn1*
39	*Pkd2*
40	*LOC100043982*
41	*Krcc1*
42	*Vamp3*
43	*M6pr*

44	*Copb1*
45	*Sec22b*
46	*Ptbp2*
47	*Sec61a1*
48	*Rab28*
49	*Spop*
50	*Ccnb1ip1*
51	*Man1a*
52	*Nit2*
53	*Rasa1*
54	*Zfp187*
55	*Ctsb*
56	*Ngly1*
57	*Mobkl1b*
58	*Fgfr2*
59	*Atxn10*
60	*Nrbp1*
61	*Bnip3*
62	*Sec61a2*
63	*Fam18b*
64	*Fbxo21*
65	*Vcl*
66	*6330409N04Rik*
67	*Papolg*
68	*Matr3*
69	*Esf1*
70	*Hsd17b12*
71	*Cpne1*
72	*Fam126a*
73	*Tmed2*
74	*Igfbp5*
75	*Zfp445*
76	*Nop58*
77	*Uba6*
78	*1200016B10Rik*
79	*Eif4e*
80	*Tax1bp3*
81	*Capn7*
82	*Gm1564*
83	*Rspo1*
84	*Tgfbr1*
85	*Notch2*
86	*Alb*
87	*Uba3*
88	*Hectd1*
89	*6330503K22Rik*

90	*Taf6*
91	*Rhox13*
92	*Fam120a*
93	*Med4*
94	*Rad17*
95	*Tex14*
96	*Usp34*
97	*Bnc1*
98	*Parg*
99	*Clns1a*
100	*Psmd1*

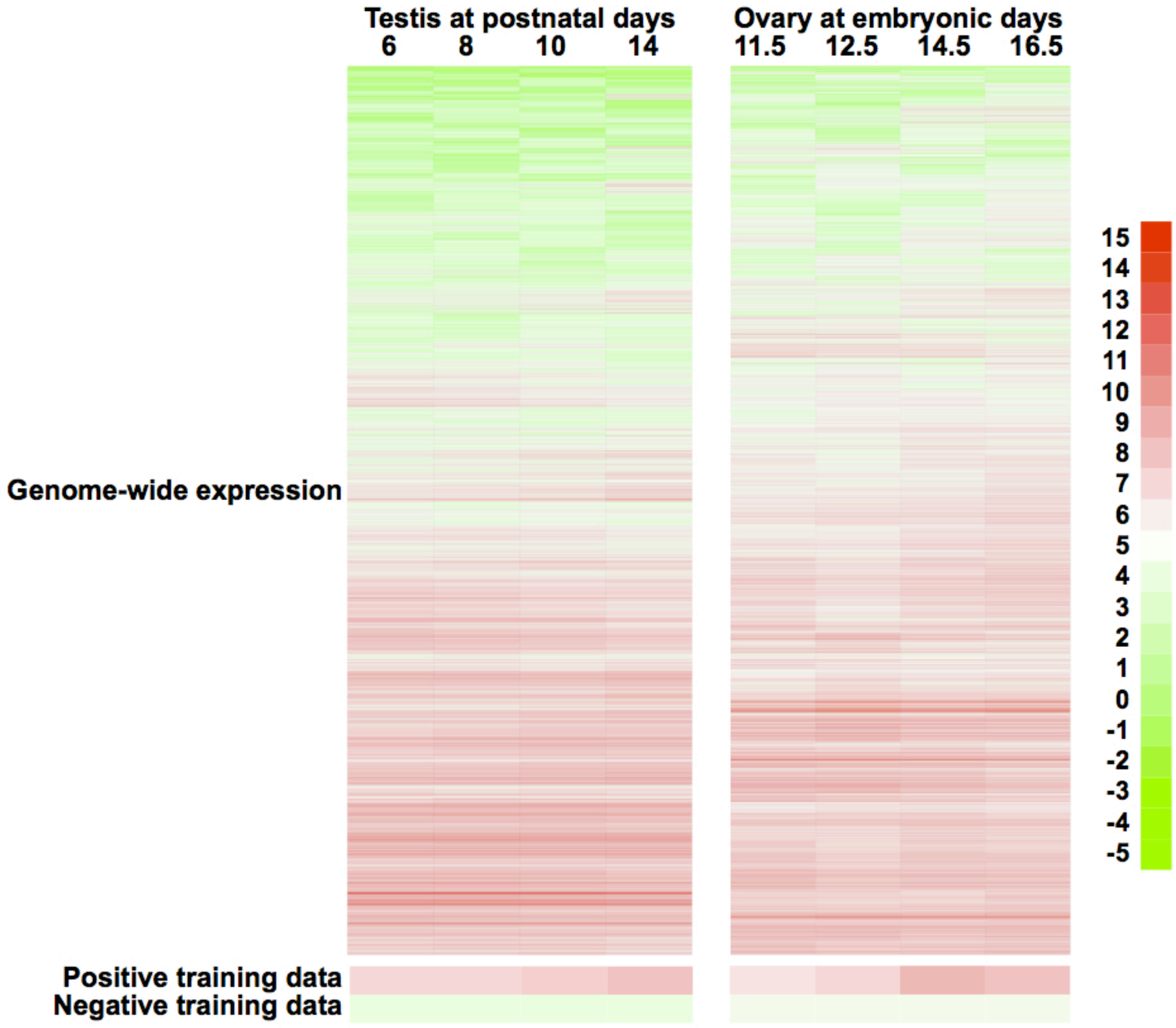

Figure S1 - Global gene expression and training data expression in meiotic prophase
Gene expression levels were log2 transformed. The global expression data were analyzed by average linkage hierarchical clustering using uncentered correlation as distance metrics. The average expression profiles of training data are shown at the bottom of the figure.
